# Investigation of antimicrobial synergism of actinomycin derivatives from *Streptomyces parvus* 35M1 against *Escherichia coli* ATCC 25922

**DOI:** 10.55730/1300-0152.2787

**Published:** 2025-11-26

**Authors:** Özge CAN, Mustafa Ünver KURT, Melis KÜÇÜKSOLAK, Ataç UZEL, Erdal BEDİR

**Affiliations:** 1 Department of Bioengineering, Faculty of Engineering, Izmir Institute of Technology, İzmir, Turkiye; 2 Department of Biology, Faculty of Science, Ege University, İzmir, Turkiye

**Keywords:** *Actinomycetes*, dactinomycin, drug synergism, *Escherichia coli*

## Abstract

**Background/aim:**

Limited drug development and increased antibiotic tolerance among Gram-negative bacteria have led researchers to consider combination therapy. Actinomycin derivatives, particularly actinomycin D, exhibit a wide range of bioactivities, including antibacterial effects. However, actinomycin D is less effective against Gram-negative pathogens. Therefore, it is essential to demonstrate the synergy of actinomycin D and its derivatives with clinically known antibiotics against *Escherichia coli* ATCC 25922 and investigate the effects on cellular morphology.

**Materials and methods:**

*Streptomyces parvus* 35M1 isolated from coastal sediment in İzmir/Türkiye was identified via genome sequencing. Large-scale (30 L) fermentation followed by chromatographic purification yielded compounds **1** (actinomycin D), **2** (actinomycin X_2_), and **3** (actinomycin X_0ß_), structurally confirmed by NMR spectroscopy. Later, checkerboard assays were used to assess combinatorial interactions with clinically relevant antibiotics in triplicate across two independent biological replicates. Synergistic interactions were evaluated in SynergyFinder using Zero Interaction Potency, Highest Single Agent, Loewe Additivity, and Bliss Independence models. Morphological alterations under synergistic treatments were examined via scanning electron microscopy.

**Results:**

Individual actinomycins exhibited weak antimicrobial activity (MICs > 100 μM). Nevertheless, strong synergistic effects were actinomycins D and X_2_ combinations with polymyxin B and kanamycin (Most Synergistic Area scores >10). Relatively high synergy scores were obtained for the actinomycin X_2_-polymyxin B combination, with values of 33.49, 32.69, 33.98, and 38.18 in the Loewe, Bliss, ZIP, and HSA models, respectively. Only actinomycin X_2_ synergized with nalidixic acid (MSAs ≥13.49 ± 2.63), while all actinomycins displayed additive/indifferent effects with rifampicin and ampicillin. Further, SEM analysis revealed cellular deformation, including elongation, membrane rupture, and dent formation.

**Conclusion:**

This work represents the first multimodel synergy analysis of actinomycin analogs against *E. coli*, underscoring their potential. However, these findings are limited to in vitro assays on a single reference strain; thus, further validation with in vivo models will be necessary.

## 1. Introduction

Natural products derived from microorganisms are essential for producing valuable pharmaceutical agents. Among these microorganisms, *Streptomyces* is the most widespread bacterial genus known for its exceptional ability to produce bioactive compounds, including clinically prescribed antibiotics (Barka et al., 2015; Gezer et al., 2022). Actinomycins are mainly chromopeptide antibiotics composed of two cyclic depsipeptides attached to a phenoxazine chromophore (Anthony and Helmut, 2011). A notable member of this class, actinomycin D is a pioneering antibiotic with antitumor activity and has been widely used in treating various pediatric tumors. From the mechanistic perspective, actinomycin D involves inserting between guanine-cytosine pairs in the minor grooves of DNA, effectively interfering with the double helix’s unwinding and inhibiting RNA polymerase activity. Thus, it functions as an inhibitor of ribosome biogenesis (Slotnick, 1960; Koba and Konopa, 2005).

Although actinomycin D has antibacterial activities against various pathogens, it cannot be used as an antibiotic due to its high hepatotoxicity and narrow therapeutic window (Tan et al., 1959). Despite the pharmacological constraints, actinomycins may have renewed therapeutic potential since limited drug development and increasing antibiotic resistance have prompted researchers to consider combination therapy (Hossain et al., 2020; Si et al., 2023). Supporting this view, El-Naggar reported that actinomycin X_2_, a 4-oxoproline derivative of actinomycin D, exhibited synergistic effects when combined with gentamicin, doxycycline, lincomycin, sulbactam/ampicillin, kanamycin, and streptomycin against drug-resistant *Staphylococcus aureus* (El-Naggar, 1998).

Gram-negative bacteria display diminished susceptibility to many known antibiotics, including actinomycin D, predominantly owing to their outer membrane (OM) and high genetic plasticity (Poirel et al., 2018; Braz et al., 2020). Since these intrinsic characteristics pose significant challenges when combating infections, the literature suggests a promising potential for using polytherapy to treat clinically relevant species (Chamberlin et al., 2019; Tian et al., 2021; Ridyard et al., 2023). Recent studies have supported the importance of combinatorial approaches against Gram-negative pathogens. For instance, Wesseling and Martin stated that polymyxin B nonapeptide, designed to disrupt the OM transiently, significantly enhanced the effectiveness of vancomycin, rifampicin, and novobiocin against strains of *Escherichia coli* and *Klebsiella pneumoniae*, achieving a FICI of ≤0.25 (Wesseling and Martin, 2022). Similarly, Wang et al. demonstrated that combinations of colistin with dalbavancin or oritavancin had bactericidal effects in clinical isolates of *E. coli* and *K. pneumoniae* (Wang et al., 2024). While several studies have demonstrated the potential of synergistic combinations in Gram-negative pathogens, the continuous emergence of diverse resistance and tolerance mechanisms and strain-specific phenotypes underscores the need to expand the range of combinatorial strategies.

In the literature, studies on actinomycin D have mainly focused on its anticancer and individual antibacterial properties, while its synergistic potential against Gram-negative bacteria remains overlooked. As part of our studies on metabolites isolated from the phylum Actinomycetota (Ozcan et al., 2015; Aksoy et al., 2016; Khan et al., 2019; Aksoy et al., 2021), the current study provides the first multi-model synergy evaluation of actinomycin D, X_2_, and X_0ß_ against *E. coli* ATCC 25922 while correlating the findings with SEM-based morphological alterations. Thus, these findings highlight the potential of actinomycins to be therapeutically repurposed against Gram-negative pathogens.

## 2. Materials and methods

### 2.1. Bacterial strains and culture conditions

The isolate was obtained from a sediment sample (ooze, clay-like) collected from the coastal area of Ilıca Bay (İzmir, Türkiye; 38°12′29″ N, 26°38′09″ E). Samples were transferred into sterile Falcon tubes, stored at 4 °C until processing, and homogenized in 0.9% (w/v) saline for 2 h at 28 °C. The suspensions were serially diluted (10^−1−^10^−2^) and spread onto M1 agar [10 g soluble starch (Isolab Laborgeräte GmbH, Cat. No. 970.012.1000), 4 g yeast extract (Biolife Italiana, Cat. No. 4122202), 2 g peptone (Biolife Italiana, Cat. No. 401278B2), 39 g.L^−1^ artificial sea salt (ReeFlowers Caledonia Sea Salt), and 20 g.L^−1^ bacteriological agar in 1 L dH_2_O, pH 7.0] supplemented with 50 μg.mL^−1^ nystatin (Sigma-Aldrich Cat. No. N6261) and 20 μg.mL^−1^ nalidixic acid (Sigma-Aldrich Cat. No. N8878), following Aksoy et al. (2016). Plates were incubated at 28 °C for 4 weeks. Actinobacteria-suspected colonies were subcultured using the streak plate method, and colony morphology was assessed based on texture, aerial and substrate mycelial color, pigment production, and sporulation characteristics.

*E. coli* ATCC 25922 was chosen as the standard model organism for antibiotic susceptibility testing per Clinical and Laboratory Standards Institute (CLSI) guidelines. The strain was grown on Tryptone Soy Agar (TSA) (Thermo Scientific Oxoid, Cat. No. RM295.00) at 37 °C overnight. For the antimicrobial assays, pure colonies were suspended in a physiological saline solution to have the McFarland 0.5 turbidity and inoculated into Mueller-Hinton Broth (MHB, Thermo Scientific Oxoid, Cat. No. CM0405B). All procedures were conducted under Biosafety Level 2 (BSL-2) containment in compliance with internationally accepted biosafety regulations and guidelines for safely handling microorganisms.

### 2.2. Whole genome sequencing and assembly

The isolate was grown in M1 broth at 28 °C and 150 rpm for 48 h. Later, the genomic DNA was extracted following the procedure of Praveen and Tripathi (2009). DNA concentration and purity were assessed using a NanoDrop 2000 spectrophotometer (Thermo Fisher Scientific, USA) and quantified with a Qubit 3.0 Fluorometer (Thermo Fisher Scientific, USA). Thereafter, shotgun sequencing and De novo assembly were performed by Gen-Era Diagnostics Inc. (Türkiye) using Illumina NovaSeq 6000. Further, the values of digital DNA-DNA hybridization (dDDH) and Average Nucleotide Identity (ANIm and ANIb) values were calculated using the genome-to-genome distance calculator (GGDC) with formula 2: identities/HSP length and JSpeciesWS online service (Richter et al., 2016; Meier-Kolthoff et al., 2022).

### 2.3. Preparative-scale fermentation, purification, and characterization of the compounds 1–3

The isolate was sporulated on Soy Flour Mannitol (SFM) agar [20 g mannitol (Merck, Cat. No. 105982), 15 g NaCl (Isolab Laborgeräte GmbH, Cat. No. 969.036), 20 g soy flour (food-grade gluten-free, Brand: Astera, Türkiye) and 15 g bacteriological agar (Isolab Laborgeräte GmbH, Cat. No. 902.016)] dissolved in 1 L dH_2_O, pH 7.0 for four days at 28 °C. After, the seed culture was prepared by incubating the spore suspension at 28 °C, 150 rpm for six days in M1 broth. The seed cultures were transferred into 5 L flasks containing 2 L M1 broth at an inoculum ratio of 5% (v/v). Then, preparative-scale fermentation was carried out at 28 °C, 150 rpm for twelve days. In total, 30 L cell-free fermentation broth was obtained via filtration. Then, solid-phase extraction using D101 macroporous adsorption resin was conducted to increase secondary metabolite yield. First, the resin (1:5, w/v) was mixed with the broth by shaking for two hours at 180 rpm to adsorb the secondary metabolites onto the resin. Later, desorption studies were performed utilizing gradient MeOH:dH_2_O mixtures (25% to 100%, increased by 25% with MeOH) and finally with acetone.

Then, the five main fractions were subjected to antimicrobial disc diffusion tests (128 μg.mL^−1^ per disc) against *E. coli* ATCC 25922. The fraction with the highest antibacterial activity was gradually subjected to normal and reversed-phase (RP-C_18_) silica gel column chromatography. The chemical structures of the pure compounds were elucidated using NMR spectroscopy (Varian Mercury plus-AS-400, ^1^H: 400 MHz, ^13^C:100 MHz). Structure visualization was created using ChemDraw version 15.1.

### 2.4. Determination of minimum inhibitory concentration (MIC)

The microdilution broth method was performed to determine MIC_90_ values of compounds 1–3 (up to 256 μg.mL^−1^) and antibiotics; nalidixic acid (≥98% purity, Sigma-Aldrich, N8878), ampicillin (High Purity grade, Bio Basic Asia Pacific, Cat. No. AB0028), rifampicin (USP grade, AFG Bioscience, C809V99), polymyxin B (purity >80% HPLC assay, Bioshop Canada, Cat. No. POL435), and kanamycin (High purity grade, Bio Basic Asia Pacific, Cat. No. KB0286) against *E. coli* ATCC 25922 in 96-well plates using MHB. CLSI standards were followed for preparing antibiotic stock solutions in DMSO (<2.5% v/v, PanReac AppliChem, Cat. No. A3672). The triplicate sterility and growth control groups were included in the tests. Final concentration of bacterial cells inside the wells was set to 2–5 × 10^6^ CFU.mL^−1^, and the plates were incubated at 35 °C for 18 h. Later, OD_600_ was measured using a microplate reader (Thermo Scientific Multiskan GO).

### 2.5. Checkerboard assay

A checkerboard synergy assay was employed to examine the combined effects of the isolated compounds with the antibiotics (nalidixic acid, ampicillin, rifampicin, polymyxin B, and kanamycin) against *E. coli* ATCC 25922 (Bellio et al., 2021). The antibiotics (from 0.125 to 2-fold of MIC) were loaded into wells in two-fold serial dilutions along the plate, while varied concentrations of the compounds (from 0.125 to 2-fold of MIC) were applied across the plate. Subsequently, the bacterial inoculum was transferred into the wells to achieve 2–5 × 10^6^ CFU.mL^−1^. The plates were incubated at 35 °C for 18 h. Later, OD_600_ value was measured using Thermo Scientific Multiskan GO. Data analysis was performed using the SynergyFinder web application (version 3.0), which enables unbiased evaluation of drug-drug interactions (Ianevski et al., 2022). Most synergistic area scores were calculated using four reference models: Bliss Independence (Bliss), Zero Interaction Potency (ZIP), Highest Single Agent (HSA), and Loewe’s Additivity.

### 2.6. Scanning electron microscopy (SEM) imaging

Midlogarithmic phase bacteria were washed twice and re-suspended in phosphate buffered saline (Sigma-Aldrich, Cat. No. P4417) to achieve McFarland 0.5. The cells were incubated at 37 °C overnight with MICs of the individual compounds only, which showed synergy, and the combinations were at Minimum Synergistic Concentrations (MSCs). Following the incubation, the bacteria were fixed with 3% glutaraldehyde (Merck, Cat. No. 104239) at 4 °C for 6 h and dehydrated with a series of ethanol (Honeywell, Cat. No. 32221) concentrations (30%, 50%, 75%, 95%, and 100%) by centrifuging at 5000 rpm for 10 min. Then, for each, 20 μL were drop-casted over a stub and dehumidified under the vacuum (Chakraborty et al., 2024). The morphological changes of *E. coli* ATCC 25922 were observed by SEM (FEI Quanta FEG 250) after gold sputter coating. The SEM technician was blinded to the experimental conditions, and the images were captured in a systematic and unbiased manner. Subsequent cell length measurements were performed using ImageJ (NIH, Bethesda, MD, USA).

### 2.7. Statistical analysis

All biological experiments were performed in triplicate and two independent biological replicates. MIC values were consistent across replicates and are reported as representative results (variation ≤ one twofold dilution). Growth inhibition percentages derived from OD_600_ measurements of the replicates were expressed as mean ± standard deviation (SD). Statistical comparisons were performed using one-way ANOVA followed by Tukey’s post-hoc test in GraphPad Prism (v9.0), and p ≤ 0.05 was considered statistically significant.

Most synergistic area scores (MSA) were interpreted as strong synergy if the scores were >10 and indifference/additive effect if the values were between −10 and 10, as indicated (Ianevski et al., 2022). SD values were obtained from SynergyFinder’s replicate analysis, where SD reflects variability in the underlying dose-response measurements per treatment. The weak synergy was defined as if at least two of the four synergy models had scores greater than 10.

For SEM analysis, three nonoverlapping fields were acquired per sample. After scale calibration, the length and width of 25 randomly selected, clearly distinguishable cells per treatment group were measured.

## 3. Results and discussion

### 3.1. Strain identification and isolation studies

**Figure 1:**
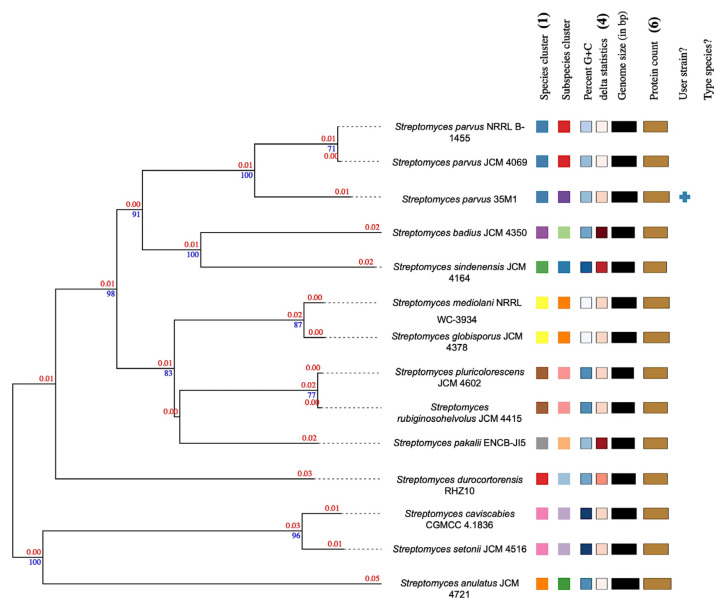
The phylogenetic tree of *S. parvus* 35M1 obtained from the Type (Strain) Genome Server (TYGS). Branch lengths are scaled according to the Genome Blast Distance Phylogeny (GBDP) distance formula D5. Numbers above the branches represent GBDP pseudo-bootstrap support values greater than 60% from 100 replications, with an average branch support of 83.8% and the tree was rooted at the midpoint. Color-coded features shown to the right of the tree represent: (1) species clusters (dDDH ≥ 70%); (2) subspecies clusters (dDDH ≥ 79%); (3) genomic G + C content (%) ranging from 57.0 to 61.2, visualized as a blue gradient from light (lower G + C) to dark blue; (4) δ-statistic values from 0.269 to 0.517, shown as a red gradient from light (lower phylogenetic divergence) to dark red; (5) genome size (bp, 4,792,107-6,307,666) represented by a gray gradient from light (smaller) to black; and (6) protein count (4419–6279) displayed as a brown gradient from light (fewer proteins) to dark brown.

A detailed investigation of colony morphology on M1 agar revealed that the strain forms light yellow aerial mycelia, as shown in Figure S1. The light microscopy imaging in Figure S2 confirmed that the strain has smooth, round spores around the long filamentous mycelia. Furthermore, the phylogenetic tree given in Figure 1 was constructed via TYGS, demonstrating that the strain is closely related to *Streptomyces parvus* NRRL B-1455. Based on the whole genome sequence, Delta (δ) statistics in the phylogenetic tree had a 0.159 δ value on average, indicating high accuracy. DNA-DNA hybridization analysis revealed that the isolate shares a dDDH value of 78.50% with *S. parvus* NRRL B-1455, supporting their classification within the same species. As shown in Table S, this relationship was further supported by ANIm and z-score values of 97.76% and 0.9991, respectively. These are consistent with the thresholds for species delineation, which are the recommended ANI (≥96.7%) and dDDH (≥70%) criteria (Hu et al., 2022). However, the isolated strain represents a novel subspecies because the probability of belonging to the same taxonomic unit as *S. parvus* NRRL B-1455 was calculated as 44.94%, below the threshold. Therefore, the strain was identified as *S. parvus* 35M1, and the sequence has been submitted to DDBJ/ENA/GenBank NCBI (Accession number: JBCLWP010000000) as a BioSample (SAMN41108085).

**Figure 2:**
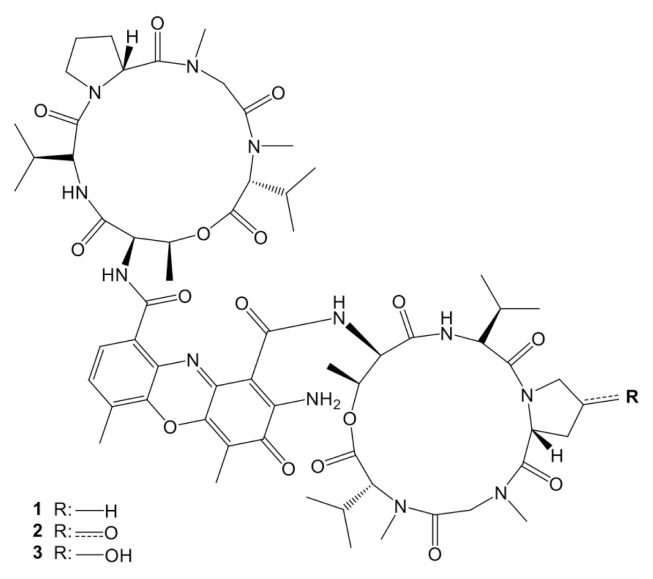
The chemical structures of the isolated actinomycins D (**1**), X_2_ (**2**), and X_0ß_ (**3**).

Before the isolation studies, the disk diffusion assay resulted in the acetone fraction having the highest zone of inhibition diameter (10 ± 1 mm) compared to other main fractions against *E. coli*. As summarized in Figure S3, further isolation afforded actinomycin D (1, 28.2 mg), X_2_ (2, 11.5 mg), and X_0ß_ (3, 10.3 mg) as red amorphous powder. The corresponding chemical structures are presented in Figure 2. The spectral data shown in Figure S4-S9 were consistent with the literature, confirming their structure (Toumatia et al., 2015; Wang et al., 2017; Qureshi et al., 2021).

### 3.2. In vitro antibacterial activities of individual compounds

Actinomycins D, X_2_, and X_0ß_ exhibited weak inhibitory effects, with MIC values of 203.9 μM (>128 μg.mL^−1^; 89.3% ± 0.56% growth inhibition), 100.8 μM (128 μg.mL^−1^; 90.3% ± 0.6%), and 201.3 μM (>128.μg.mL^−1^; 89.65% ± 0.71%), respectively. In contrast, reference antibiotics displayed stronger activity, with nalidixic acid (8.61 μM; 90.67% ± 0.6%), ampicillin (2.86 μM; 92.0% ± 0.4%), rifampicin (4.86 μM; 91.5% ± 0.5%), polymyxin B (3.07 μM; 92.3% ± 0.31%), and kanamycin (4.12 μM; 93.3% ± 0.6%). Research indicated that five actinomycins (including actinomycin D, X_2,_ and X_0ß_) demonstrated weak activity against *E. coli* and *K. pneumoniae* (MICs >128 μg.mL^−1^) (Machushynets et al., 2022). The weak activity observed against *E. coli* may be attributed to the poor permeability of actinomycin D through the OM, which is known to act as a major barrier (MacAlister et al., 1972). This finding is supported by studies showing that disruption of the OM, such as via EDTA treatment and cell wall mutations affecting integrity, enhances susceptibility to actinomycin D (Sekiguchi and Iida, 1967; Leive, 1968). Despite its weak antibacterial activity, actinomycin D has been shown to inhibit FtsZ polymerization and transcription in *E. coli*, suggesting a possible adjuvant role (Charoenwiwattanakij et al., 2020).

Regardless of the bacterial strain, further studies reported that actinomycin X_2_ is more potent than actinomycin D and X_0ß_ derivatives. In one study, actinomycin X_2_ had approximately 4-fold higher activity than actinomycin D against *S. aureus* ATCC 29213, *Staphylococcus saprophyticus* ATCC 43867, and *Staphylococcus epidermidis* ATCC 12228 (Qureshi et al., 2021). Similarly, another research reported that actinomycin X_2_ showed strong antimicrobial activity against various Gram-positive pathogens, with MIC values as low as 0.04 μM. In comparison, actinomycin X_0ß_ consistently exhibited weaker inhibition (MICs up to 2.5 μM) (Wang et al., 2017). Although these reports did not focus on the molecular basis for this reduced activity, structure-activity relationship (SAR) analyses of actinomycin derivatives indicate that modifications within the cyclic peptide rings (e.g., substitutions involving proline, oxoproline, or threonine residues) could change DNA intercalation affinity and/or affect the cellular uptake (Meienhofer and Atherton, 1973; Gallego et al., 1997; Koba and Konopa, 2005; Bitzer et al., 2006; Lo et al., 2013).

### 3.3. Synergistic effects of actinomycins-antibiotics combinations

Based on the given results, we subsequently assessed the combinatorial interactions of the actinomycins with clinically relevant antibiotics commonly used against Gram-negative pathogens. Accordingly, synergy scores were calculated using SynergyFinder after the checkerboard assay based on four established models: Loewe additivity, Bliss independence, HSA, and ZIP. In the literature, the synergistic activities of antibacterial compounds are evaluated using various statistical models, among which the fractional inhibitory concentration (FIC) index is one of the most widely used models. However, the scores obtained from FIC vary significantly depending on the bacterial strains and the experimental media (Maset et al., 2023; Fatsis-Kavalopoulos et al., 2024). Thus, statistical alternatives were used to analyze the pairwise interactions and gain a comprehensive investigation of the combination dynamics. Although synergy scores regarding the abovementioned models were computed using the SynergyFinder tool (Ianevski et al., 2022), as listed in Table, we emphasized Bliss Independence and ZIP values in this study since they are closely aligned with clinical applicability. These two models evaluate drug interactions based on probabilistic and dose-response relationships under noninteractive assumptions, while Loewe’s additivity and HSA assess synergy relative to either the additive effects or the maximal activity of individual agents (Yadav et al., 2015; Vlot et al., 2019). HSA could overestimate synergy when compounds have distinct mechanisms of action, and Loewe’s additivity assumes shared mechanisms have differing potency. Therefore, agreement between models increases confidence that the observed interaction reflects the biological effect. To facilitate interpretation given in Table, drug interaction outcomes were classified as strongly synergistic when all four models exceeded the threshold, weakly synergistic when at least two models out of four agreed to synergy. This approach provided balanced classification, reducing the likelihood of false-positive synergy calls while avoiding overly stringent criteria that could mask meaningful interactions. Hence, we report one weakly synergistic interaction between actinomycin D and nalidixic acid, with MSA scores of 21.39 (Bliss), 8.53 (ZIP), 8.82 (HSA), and 15.1 (Loewe). Notably, the actinomycin D-nalidixic acid pair achieved 97.81% ± 0.68% growth inhibition at 50.97 μM and 4.30 μM, respectively.

**Figure 3:**
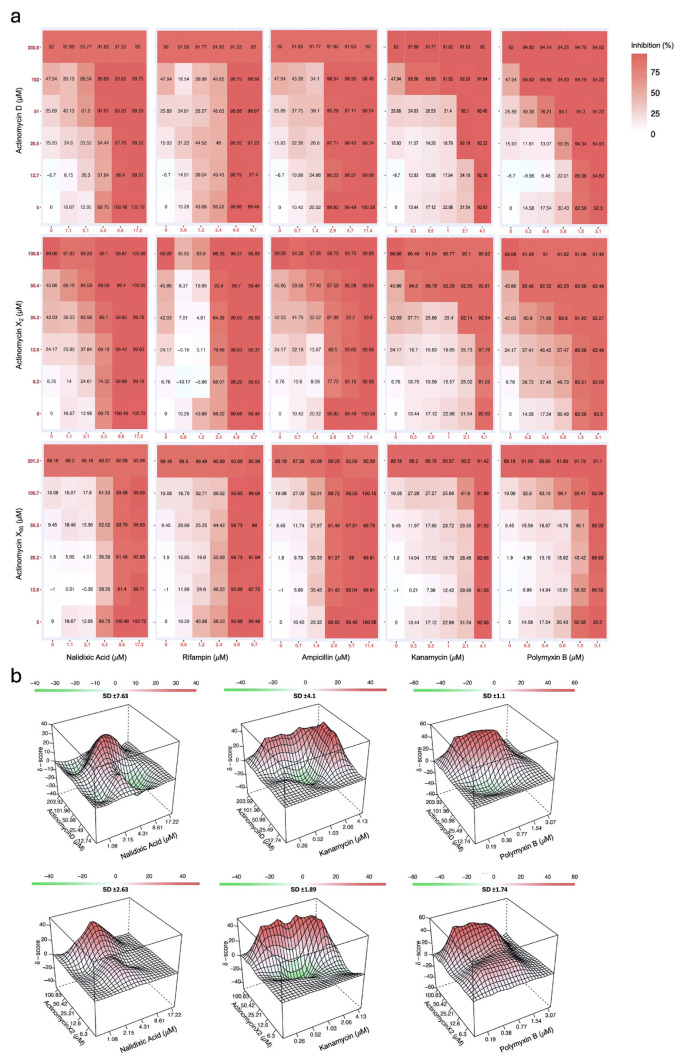
Synergistic interactions of actinomycin derivatives with antibiotics against *E. coli* ATCC 25922. a) The dose-response curves of combinations of actinomycins D, X_2_, and X_0ß_ with nalidixic acid, rifampicin, ampicillin, kanamycin, and polymyxin B, demonstrating shifts in the growth inhibition percentages, b) 3D interaction surfaces of synergistic combinations visualized under the Bliss independence model. The Standard Deviation (±SD) values indicate variability in the summary synergy score across replicate dose-response matrices, reported as one value per pair. The interaction degree is color-coded from strong antagonism (green; δ- score -40) to strong synergy (red; δ- score +40), with white gridlines marking additivity. Statistical significance was determined by one-way ANOVA as p < 0.05 vs. untreated control.

Both actinomycin D and actinomycin X_2_ exhibited strong synergistic effects when combined with kanamycin and polymyxin B, with synergy scores exceeding the threshold across all four reference models. As shown in Figure 3a, the MIC_90_ of actinomycin D decreased from 203.91 μM to 50.97 μM when combined with kanamycin, which also showed a two-fold reduction in MIC from 4.12 μM to 2.06 μM, achieving 92.1% ± 1.74% growth inhibition. Coadministration with polymyxin B resulted in synergistic interactions for both actinomycin D (Bliss MSA: 27.06; ZIP MSA: 29.50) with corresponding 4-fold MIC reductions, given in Table. Furthermore, Figure 3b represents these interactions via three-dimensional (3D) synergy landscape plots, which were generated for each synergistic drug pair to highlight the regions having synergism. The combination of actinomycin X_2_ and kanamycin yielded MSCs at 25.20 μM and 2.06 μM, respectively, achieving 92.14% ± 0.29% growth inhibition (Bliss MSA: 13.75; ZIP MSA: 14.18). The observed complementary interaction may result from inhibition of the transcription via actinomycins, combined with kanamycin’s disruption of protein synthesis, leading to enhanced effects. Similarly, actinomycin X_2_ combined with polymyxin B led to a four-fold reduction in MIC values, accompanied by 89.6% ± 1.91% growth inhibition (Bliss MSA: 32.69; ZIP MSA: 33.98). In the literature, researchers evaluated fosfomycin-based combinations against varied *E. coli* strains using the ZIP model via SynergyFinder. They reported that the fosfomycin-glycerol pair had synergy with a ZIP score of 16.4, while the fosfomycin-glycerol-glucose-6-phosphate triple combination showed no synergy with a score of 6.8 against *E. coli* ATCC 25922 (Ortiz-Padilla et al., 2022). A recent study demonstrated that benzalkonium chloride (BAC) in combination with chlorhexidine (CHX) or cetylpyridinium chloride (CPC) exhibited synergy against *E. coli* ATCC 25922 when analyzed using the Bliss and Loewe models in Combenefit. Specifically, the BAC + CHX combination yielded a Bliss synergy score of 11.2 and a Loewe score of 0.4, while BAC + CPC produced a Bliss score of 10.7 with a Loewe score of 0.2 (Greger et al., 2025). In the current study, despite high MICs of actinomycins D and X_2_, the strong synergism with low-dose polymyxin B/kanamycin suggests a translational potential. However, strain-specific SAR studies with more analogs are warranted to make final deductions.

Interestingly, only actinomycin X_2_ exhibited synergy with nalidixic acid, achieving synergy scores of 13.49 (Bliss) and 17.76 (ZIP). Nalidixic acid, a first-generation quinolone targeting DNA gyrase, typically exhibits synergy when paired with agents that either increase intracellular drug accumulation or impose complementary stress on DNA replication and repair pathways. In the literature, nalidixic acid and tetracycline displayed synergy against multidrug resistant *Acinetobacter baumannii* (FICI 0.25) and *E. coli* isolates (FICI 0.25–0.5). Thus, the researchers hypothesized that nalidixic acid contributed synergy by promoting membrane permeabilization and suppressing efflux, leading to elevated intracellular levels of tetracycline (Gaurav et al., 2021). Therefore, nalidixic acid likely enhances the intracellular accumulation of actinomycins by perturbing the outer membrane and reducing efflux. Once internalized, and consistent with relative MIC values, actinomycin X_2_ is expected to retain higher antibacterial activity at increased intracellular concentrations compared to actinomycin D and X_0ß_. Accordingly, the lack of synergy observed for actinomycin X_0ß_ may be attributed to its hydroxyproline-containing chromopeptide ring, which might reduce DNA-binding affinity and/or increase metabolic turnover.

In contrast, six combinations (actinomycins D and X_2_ with ampicillin and rifampicin, and actinomycin X_0ß_ with ampicillin and nalidixic acid) were classified as additive/indifferent, indicating their combined effect is equal to the sum of the individual effects, without enhancing or diminishing each other’s activity. The literature highlights that rifampicin’s synergy is often attributed to its combination with membrane-disrupting agents such as daptomycin and polymyxins, enhancing cellular uptake (Rand and Houck, 2004). Since actinomycins lack known membrane-disrupting properties, it does not likely improve the uptake of rifampicin, explaining our results. Similarly, the transcriptional inhibition ability of actinomycins, delaying cell division, may negatively impact the efficacy of ampicillin, which primarily targets the actively dividing cells.

### 3.4. Effect of synergy on the bacterial cell membrane

**Figure 4:**
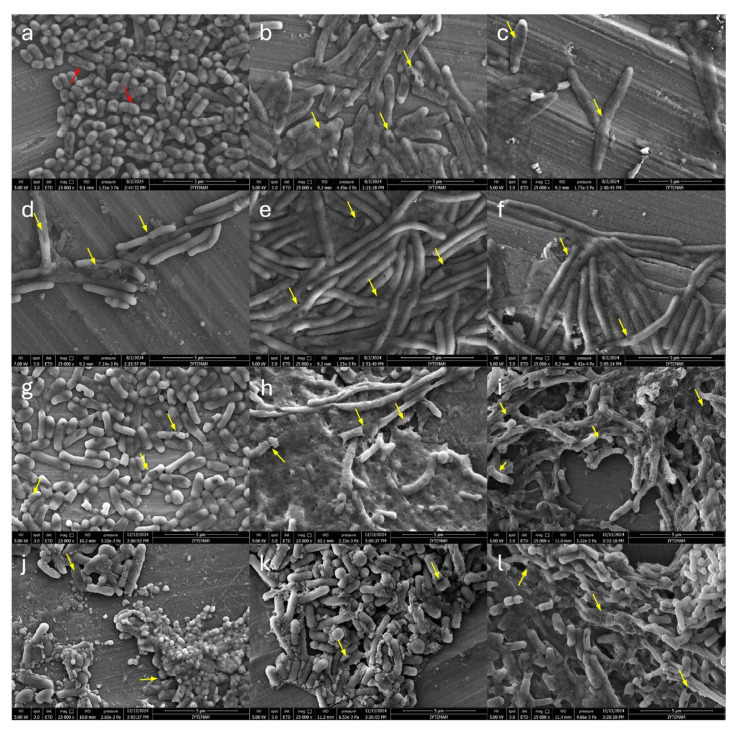
The morphology changes of *E. coli* at 25,000 × magnification in SEM upon the individual treatment at MICs and the combinations at Minimum Synergistic Concentrations (MSCs); a) vehicle control, b) actinomycin D, c) actinomycin X_2_, d) nalidixic acid, e) combination of actinomycin D: nalidixic acid, f) combination of actinomycin X_2_: nalidixic acid, g) polymyxin B, h) combination of actinomycin D: polymyxin B, i) combination of actinomycin X_2_: polymyxin B, j) kanamycin, k) combination of actinomycin D: kanamycin, l) combination of actinomycin X_2_: kanamycin. Red arrows indicate septa of binary fission, while yellow arrows highlight disrupted or deformed cells. Scale bars (5 μm) are shown at the lower right of each panel.

To evaluate the effect of synergism, the cells treated with the antibacterial combinations were examined under SEM, as given in Figure 4 and Figure S10. The vehicle control group (DMSO) was used to confirm *E. coli* ATCC 25922 morphologies, and cells appeared intact, plump, regular, and rod-shaped with approximately 1.5 ± 0.2 μm length and 0.5 ± 0.0 μm radius. By comparison, significant differences in morphology were observed with the treatment groups, including changes in cell surface integrity, surface depression, and cell elongation/filamentation. Bacterial binary fissions, in Figure 4a, are indicated with red-colored arrows. After the cells were treated with actinomycin D, the average cell elongation was 4 ± 1.63 μm in length, and dent formations were observed on the surface, as shown in Figure 4b. When the cells were treated with compound 2, *E. coli* had a deformed membrane architecture, as shown in Figure 4c, and its cell elongation became about 8 ± 2.0 μm length and 0.670 ± 0.1 μm width. However, the single treatment of nalidixic acid showed increased cellular length up to 6 ± 1.9 μm with an average of 0.701 ± 0.1 μm width according to Figure 4d. The synergy between nalidixic acid and actinomycin D resulted in visible pores on the surface and an average of 18.25 ± 2.56 μm length, shown in Figure 4e. Meanwhile, after nalidixic acid was combined with actinomycin X_2_, drastic filamentation was observed, with a cellular length up to 24.64 ± 3.83 μm. *E. coli* cells became long and rope-like without binary fissions, as presented in Figure 4f. The findings align with the literature because nalidixic acid leads to replication arrest and cell elongation by inducing the SOS and/or UV-inducible response pathways in *E. coli* at subinhibitory concentrations (Chadha and Khullar, 2021).

Nonetheless, the combinations of actinomycins D and X_2_ with polymyxin B caused notably more severe damage to morphology, as seen in Figures 4h–4j. As previously reported, polymyxin B has been shown to enhance the efficacy of traditional antibiotics (Elemam et al., 2010; Liu et al., 2020; Wesseling and Martin, 2022). In this context, its ability to disrupt bacterial membrane integrity likely facilitates increased intracellular uptake of actinomycin D and X_2_, thereby enhancing their activity per concentration.

In contrast, when the cells were treated with kanamycin alone, they exhibited surface roughening and blebbing with extracellular debris. Among the treatment groups, the co-treatment of actinomycin D with kanamycin resulted in relatively minimal cell elongation, as demonstrated in Figure 4k. However, a direct comparison of cell elongation between the polymyxin B and kanamycin combination groups cannot be made, as the cells were aggregated and overlapped, preventing accurate measurement of individual cell lengths.

Despite the promising synergistic effects observed in vitro, the clinical translation of actinomycin derivatives is hindered by the challenge of high hepatotoxicity (Tan et al., 1959). Nevertheless, due to its large peptide structure, drug-delivery strategies such as nanoparticle encapsulation and controlled delivery systems have been proposed to improve its safety (Jarmila P et al., 2024; Zimowska K et al., 2024). Furthermore, structural optimization or semisynthetic modification (e.g., altering peptide side chains or substituting chromophores) has been reported to improve pharmacokinetic profiles with lower acute toxicity (Zhang et al., 2010). Indeed, several FDA-approved combination products, such as liposomal daunorubicin/cytarabine (Lancet et al., 2018), pegylated liposomal doxorubicin with bortezomib (Sonneveld et al., 2008), and liposomal amphotericin B (Stone et al., 2016) have demonstrated that co-formulation or encapsulation can substantially mitigate toxicity while preserving synergistic efficacy. These clinical precedents indicate that similar formulation approaches could feasibly translate actinomycin combinations into safe therapeutic options.

In conclusion, this work presents the first multi-model comparative synergy evaluation of actinomycin D, X_2_, and X_0ß_, aligning with the current interest in the literature on combinatorial therapy to address escalating antimicrobial resistance and tolerance. Accordingly, although these findings highlight the potentiation of actinomycins, further studies involving diverse clinical isolates, expanded combinatorial panels, and in vivo models are essential to translate this approach.

## Supplementary Data

Figure S1 The colony morphology of *S. parvus* 35M1.

Figure S2 The light microscope (Olympus) image of *S. parvus* 35M1 at 100 × magnification.

Figure S3 The bioactivity-guided isolation scheme of actinomycin derivatives.

Figure S4 The 1H NMR spectrum of Compound **1** (in CDCl3, 1H: 400 MHz, 13C:100 MHz).

Figure S5 The 13C NMR spectrum of Compound **1** (in CDCl3, 1H: 400 MHz, 13C:100 MHz).

Figure S6 The 1H NMR spectrum of Compound **2** (in CDCl3,1H: 400 MHz, 13C:100 MHz).

Figure S7 The 1H NMR spectrum of Compound **3** (in CDCl3, 1H: 400 MHz, 13C:100 MHz).

Figure S8 The COSY spectrum of Compound **1** (in CDCl3, 1H: 400 MHz, 13C:100 MHz).

Figure S9 The HMBC spectrum of Compound **1** (in CDCl3, 1H: 400 MHz, 13C:100 MHz).

Figure S10 The changes in the morphology of *E. coli* at 5000 × magnification in SEM upon the individual treatment at MICs and combinations at MSCs; a) vehicle control, b) actinomycin D, c) actinomycin X_2_, d) nalidixic acid, e) combination of actinomycin D: nalidixic acid, f) combination of actinomycin X_2_: nalidixic acid, g) polymyxin B, h) combination of actinomycin D: polymyxin B, i) combination of actinomycin X_2_: polymyxin B, j) kanamycin, k) combination of actinomycin D: kanamycin, l) combination of actinomycin X_2_: kanamycin. Scale bars (30 μm) are shown at the lower right of each panel.

**Table S:** GGDC (with formula 2: identities / HSP length) and JSpeciesWS results belonging to *S. parvus* 35M1.

Genome	*S. parvus* 35M1	*S. glob sporus* C-1027	*S. pakal* ENCB-J15	*S. parvus* NRRL B-1455	*S. glob sporus* JCM 4378	*S. bad us* JCM 4350	*S. parvus* JCM 4069
**Access on number**	JBCLWP010000000	CP013738.1	JARWAF000000000.1	VXCD00000000	GCA_014649555.1	BMSZ00000000.1	GCA_014648875
**DDH estimate (GLM-based) (%)**	-	55.60	56.10	78.50	55.50	55.00	78.00
**Probability DDH (same species) (%)**	-	36.93	38.81	89.36	36.7	35.03	88.92
**Probability DDH (same subspecies) (%)**	-	8.11	8.63	44.94	8.05	7.62	35.57
**Model C.I.**	-	52.8–58.3	53.3–58.8	75.5–81.2	52.8–58.2	52.3–57.7	75.1–80.7
**D stance**	-	0.0598	0.0588	0.0251	0.0599	0.0609	0.0257
**G-C difference (%)**	-	0.02	0.01	0.04	0.18	0.05	0.00
**ANIb (%)**	-	93.22	93.03	96.15	93.04	92.43	96.12
**ANIm (%)**	-	94.46	94.52	97.76	94.38	94.34	97.70
**Aligned (%)**	-	75.04	73.60	80.02	74.81	72.79	80.56
**Z-score (from TCS)**	-	0.999	0.99908	0.99911	0.99914	0.9992	0.99929

## Figures and Tables

**Table:** The MIC values of individual agents and MSCs of the combinations were listed together with the MSA scores of four reference models, calculated by SynergyFinder using the dose-response matrices.

Combinations	MIC (μM)		% Inhibition (± SD)	MSAs				Interp.
	
	Alone	Combined		Loewe’s Additivity	Bliss	ZIP	HSA	
Actinomycin D	203.91	50.97	97.81 ± 0.68	21.39	8.53	8.82	15.1	Weak synergy
Nalidixic acid	8.61	4.3						

Actinomycin X_2_	100.83	25.2	89.1 ± 0.6	22.3	13.49	14.55	17.76	Synergy
Nalidixic acid	8.61	4.3						

Actinomycin X_0ß_	201.34	100.67	93.08± 0.25	−0.54	−2.84	−1.81	−0.15	Additive
Nalidixic acid	8.61	8.61						

Actinomycin D	203.91	101.95	98.75 ± 2.1	6.2	0.38	−0.92	1.73	Additive
Rifampicin	4.86	4.86						

Actinomycin X_2_	100.83	50.41	93.41 ± 0.8	2.44	4.98	3.66	−3.9	Additive
Rifampicin	4.86	2.43						

Actinomycin X_0ß_	201.34	201.34	93.09 ± 0.7	-	-	-	-	No change*
Rifampicin	4.86	4.86						

Actinomycin D	203.91	101.95	98.34 ± 1.2	−1.27	3.37	2.02	7.19	Additive
Ampicillin	2.86	2.86						

Actinomycin X_2_	100.83	100.83	97.96 ± 0.57	11.36	4.8	5.46	6.68	Additive
Ampicillin	2.86	1.43						

Actinomycin X_0ß_	201.34	100.67	88.72 ± 2.6	2.21	−3.72	−2.63	3.62	Additive
Ampicillin	2.86	2.86						

Actinomycin D	203.91	25.48	92.1 ± 1.74	33.48	17.64	18.92	28.04	Synergy
Kanamycin	4.12	2.06						

Actinomycin X_2_	100.83	25.2	92.14 ± 0.29	29.9	13.75	14.18	20.51	Synergy
Kanamycin	4.12	2.06						

Actinomycin X_0ß_	201.34	201.34	91.42 ± 0.9	-	-	-	-	No change*
Kanamycin	4.12	4.12						

Actinomycin D	203.91	50.97	94.1 ± 0.43	40.41	27.06	29.5	37.74	Synergy
Polymyxin B	3.07	0.76						

Actinomycin X_2_	100.83	25.2	89.6 ± 1.91	33.49	32.69	33.98	38.18	Synergy
Polymyxin B	3.07	0.76						

Actinomycin X_0ß_	201.34	201.34	91.1 ± 0.67	-	-	-	-	No change*
Polymyxin B	3.07	3.07						

* MSA scores were not determined if all the MIC and MSC values remained unchanged.
