# Supplementary figures and images for: Investigation of antimicrobial synergism of actinomycin derivatives from Streptomyces parvus 35M1 against Escherichia coli ATCC 25922
Source: Turk J Biol. 2025 Nov 26;50(1):17–28. doi: 10.55730/1300-0152.2787 (PMC12978764; doi:10.55730/1300-0152.2787)

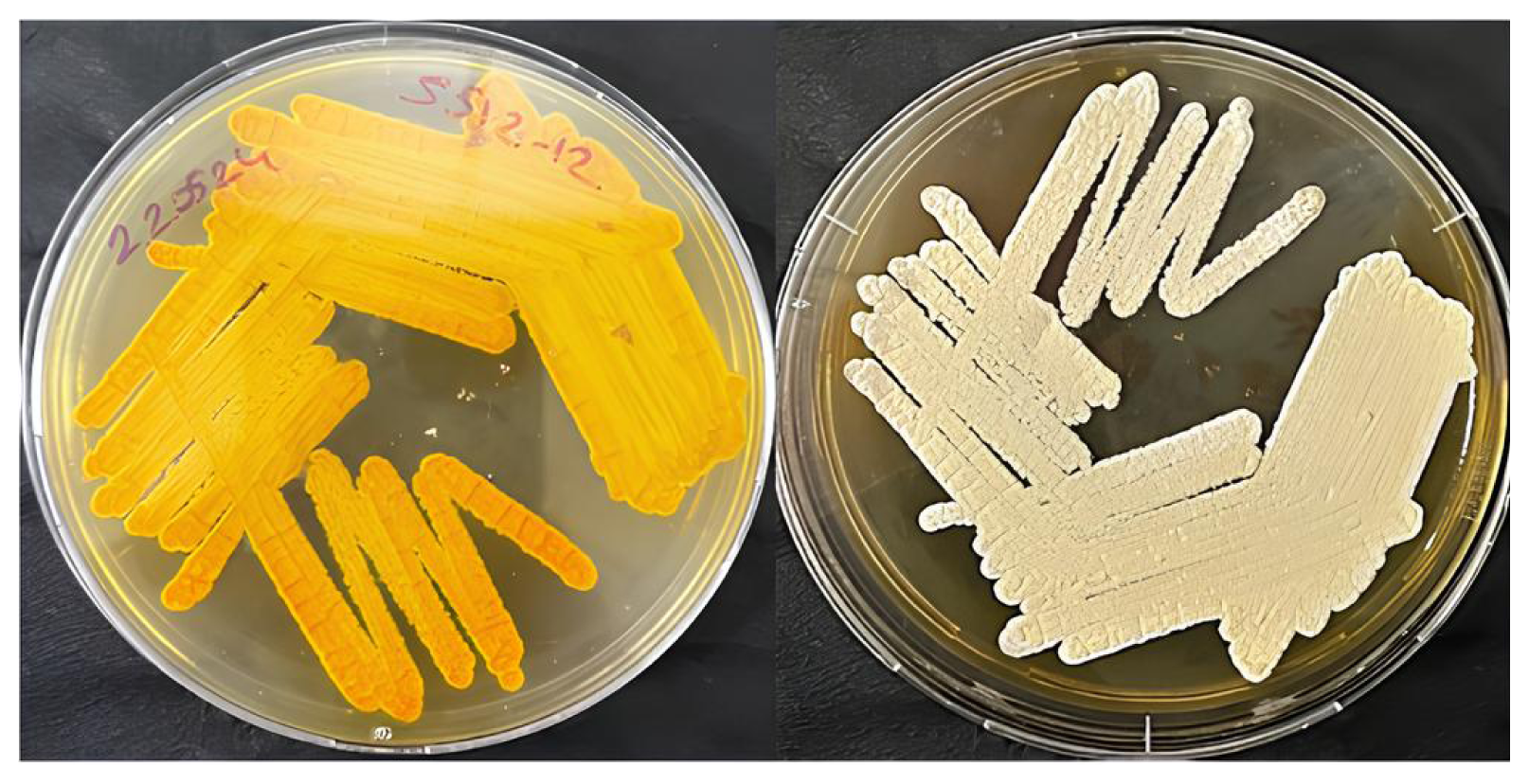

Supplement: Figure S1 — The colony morphology of S. parvus 35M1. [file tjb-50-01-17s1.tif]

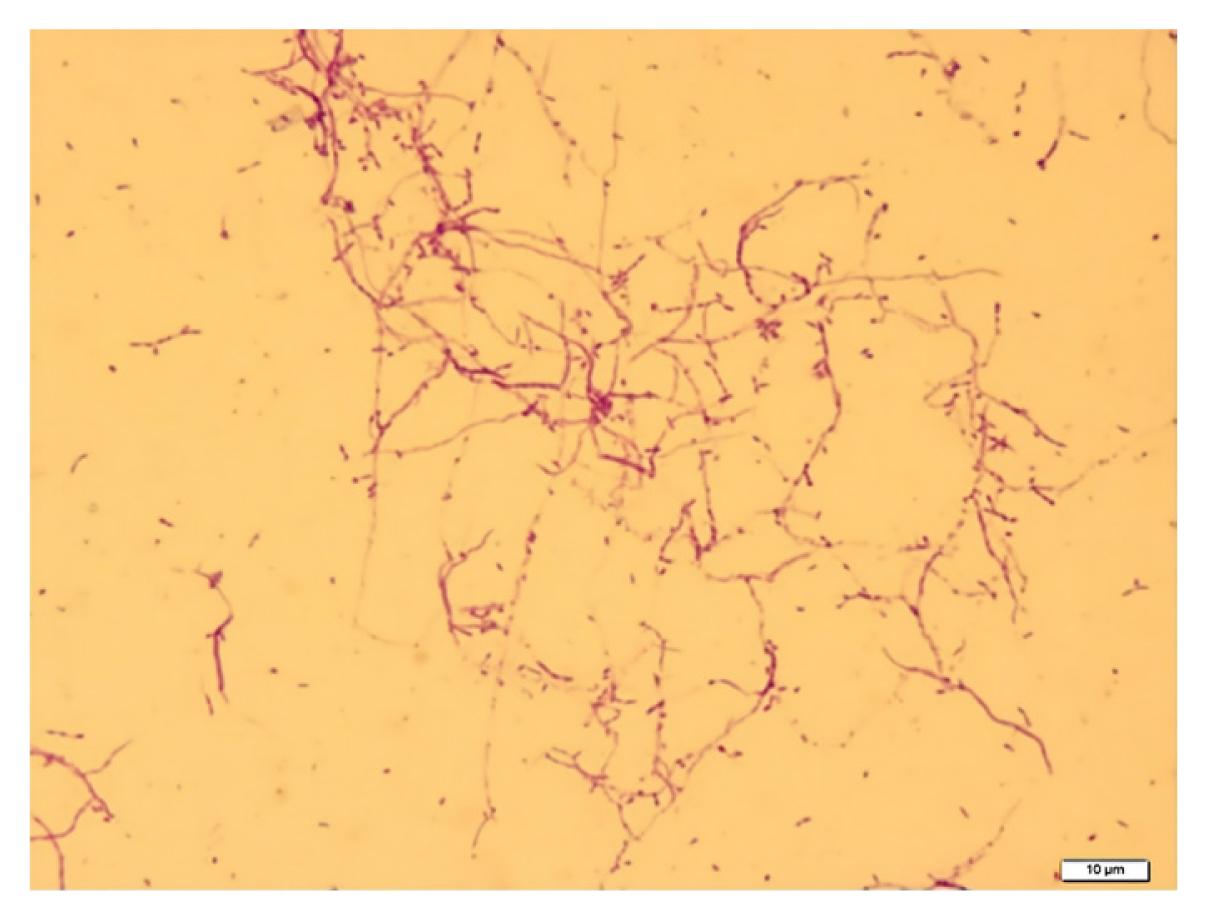

Supplement: Figure S2 — The light microscope (Olympus) image of S. parvus 35M1 at 100 × magnification. [file tjb-50-01-17s2.tif]

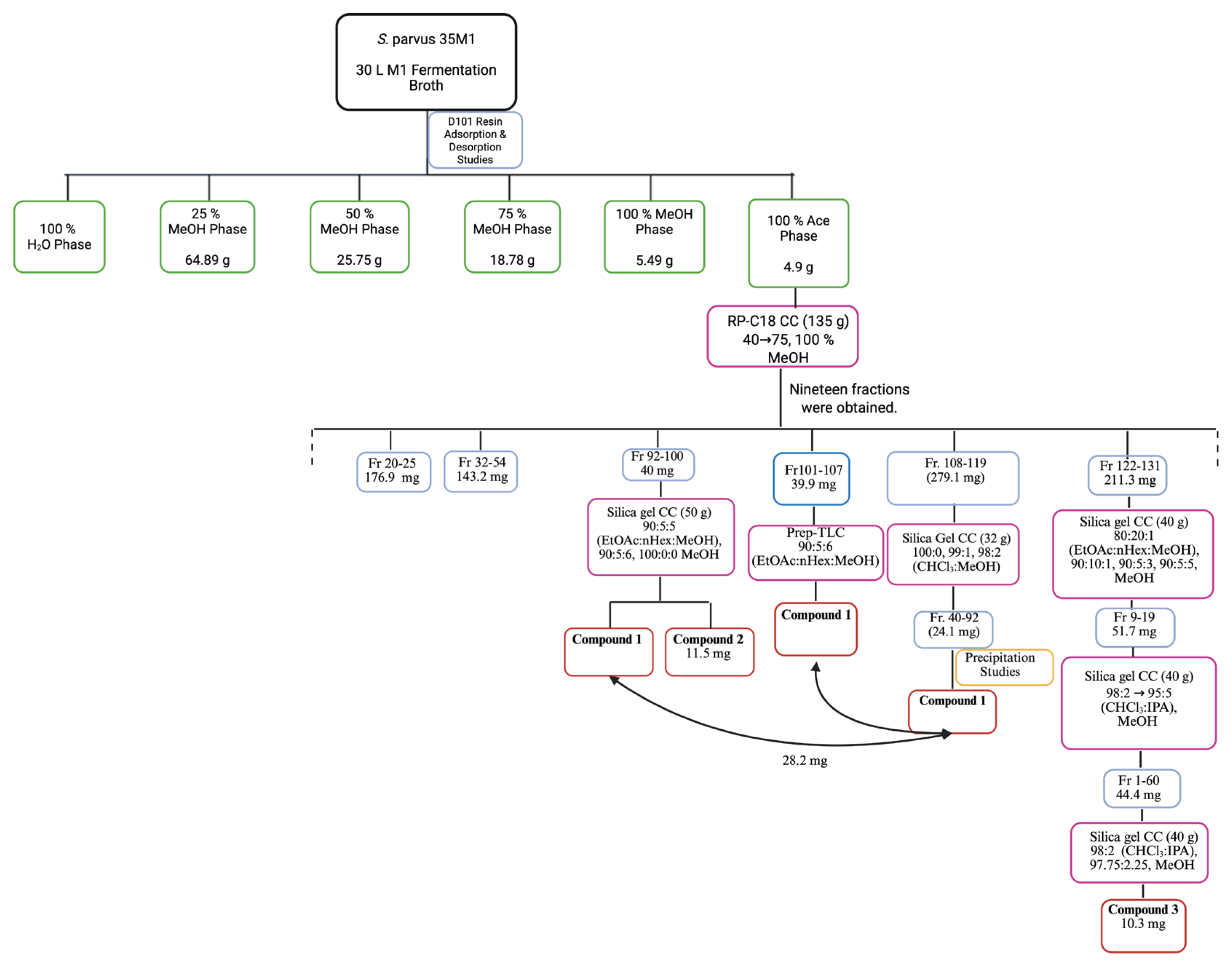

Supplement: Figure S3 — The bioactivity-guided isolation scheme of actinomycin derivatives. [file tjb-50-01-17s3.tif]

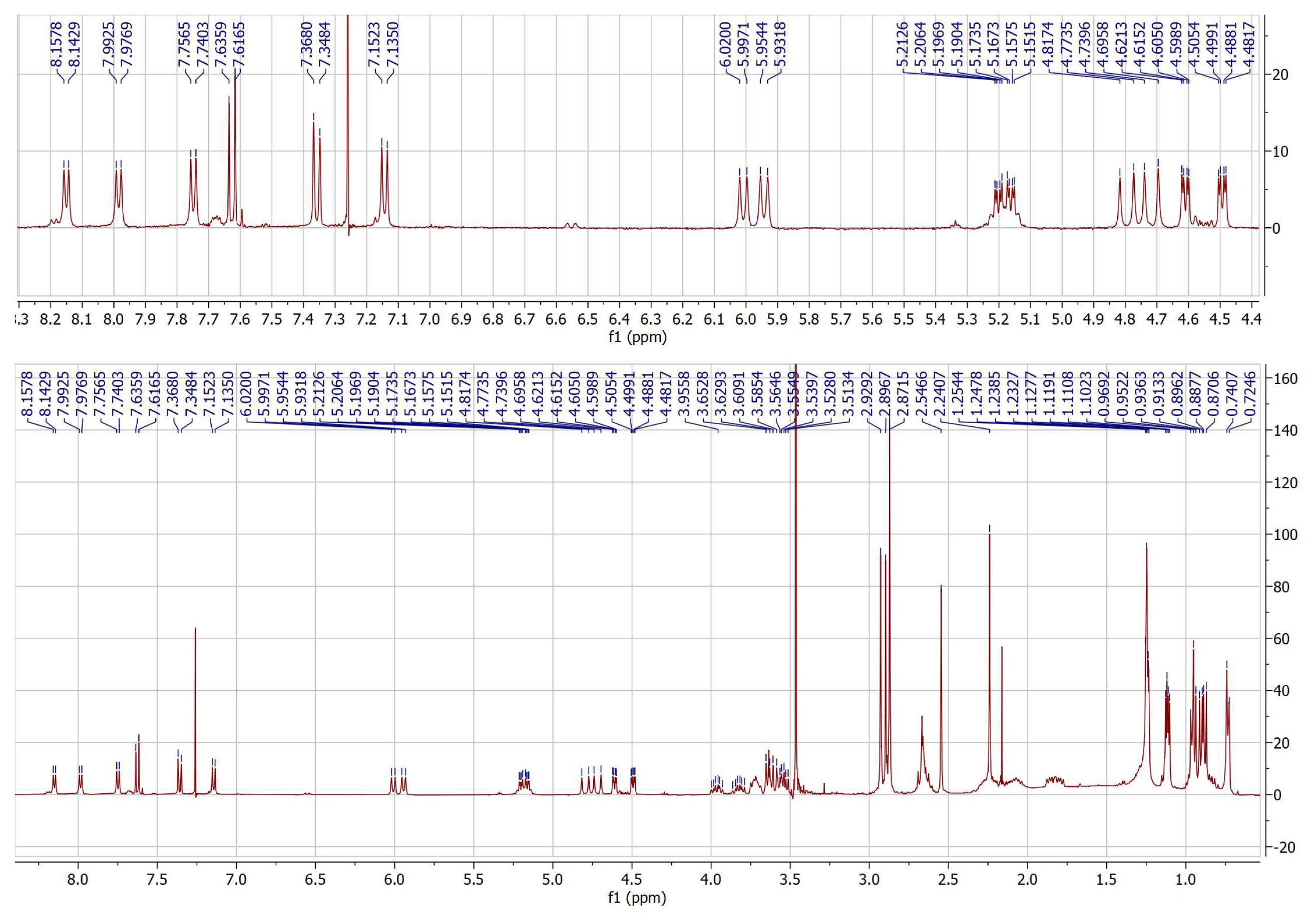

Supplement: Figure S4 — The 1H NMR spectrum of Compound 1 (in CDCl3, 1H: 400 MHz, 13C:100 MHz). [file tjb-50-01-17s4.tif]

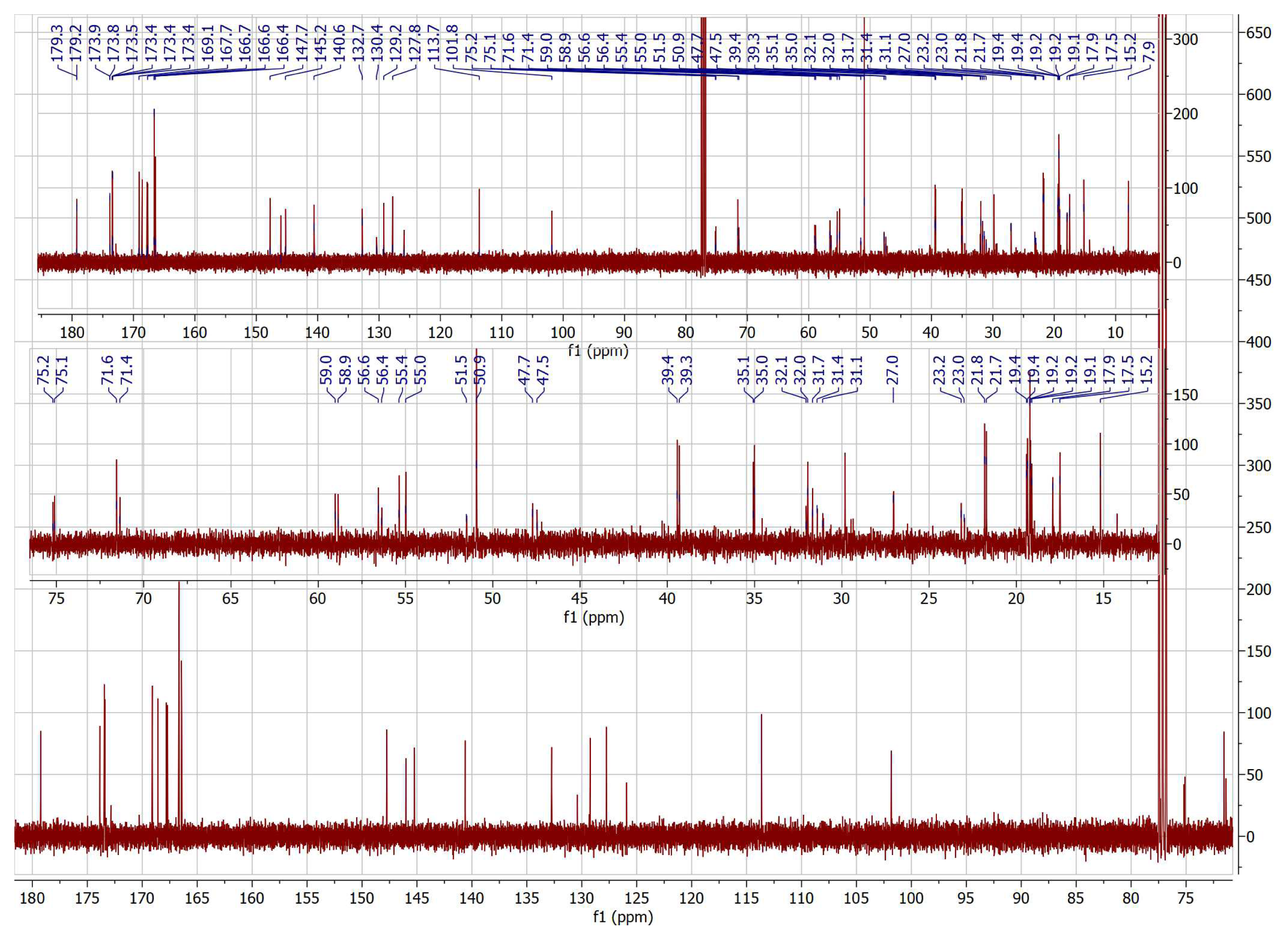

Supplement: Figure S5 — The 13C NMR spectrum of Compound 1 (in CDCl3, 1H: 400 MHz, 13C:100 MHz). [file tjb-50-01-17s5.tif]

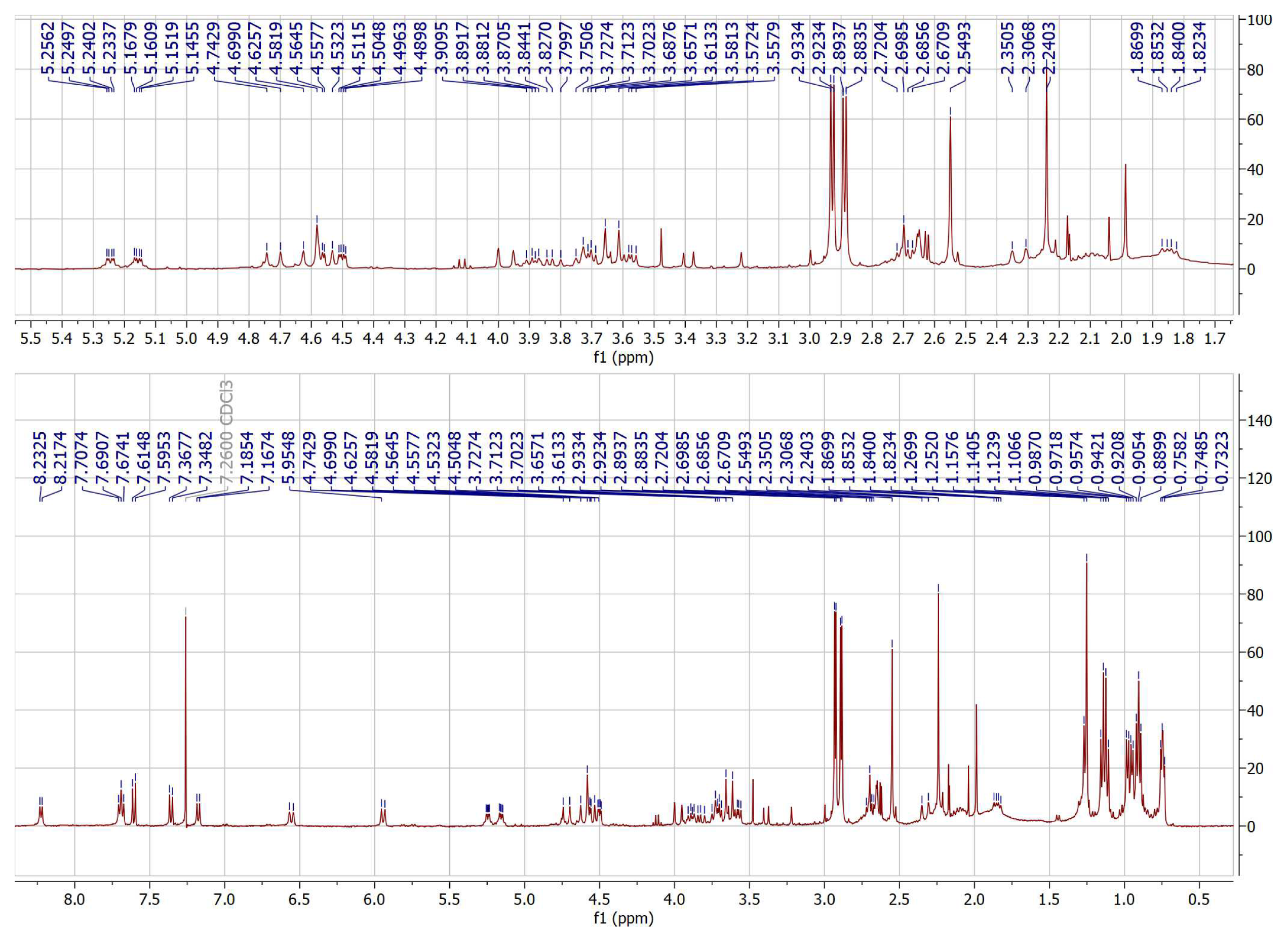

Supplement: Figure S6 — The 1H NMR spectrum of Compound 2 (in CDCl3,1H: 400 MHz, 13C:100 MHz). [file tjb-50-01-17s6.tif]

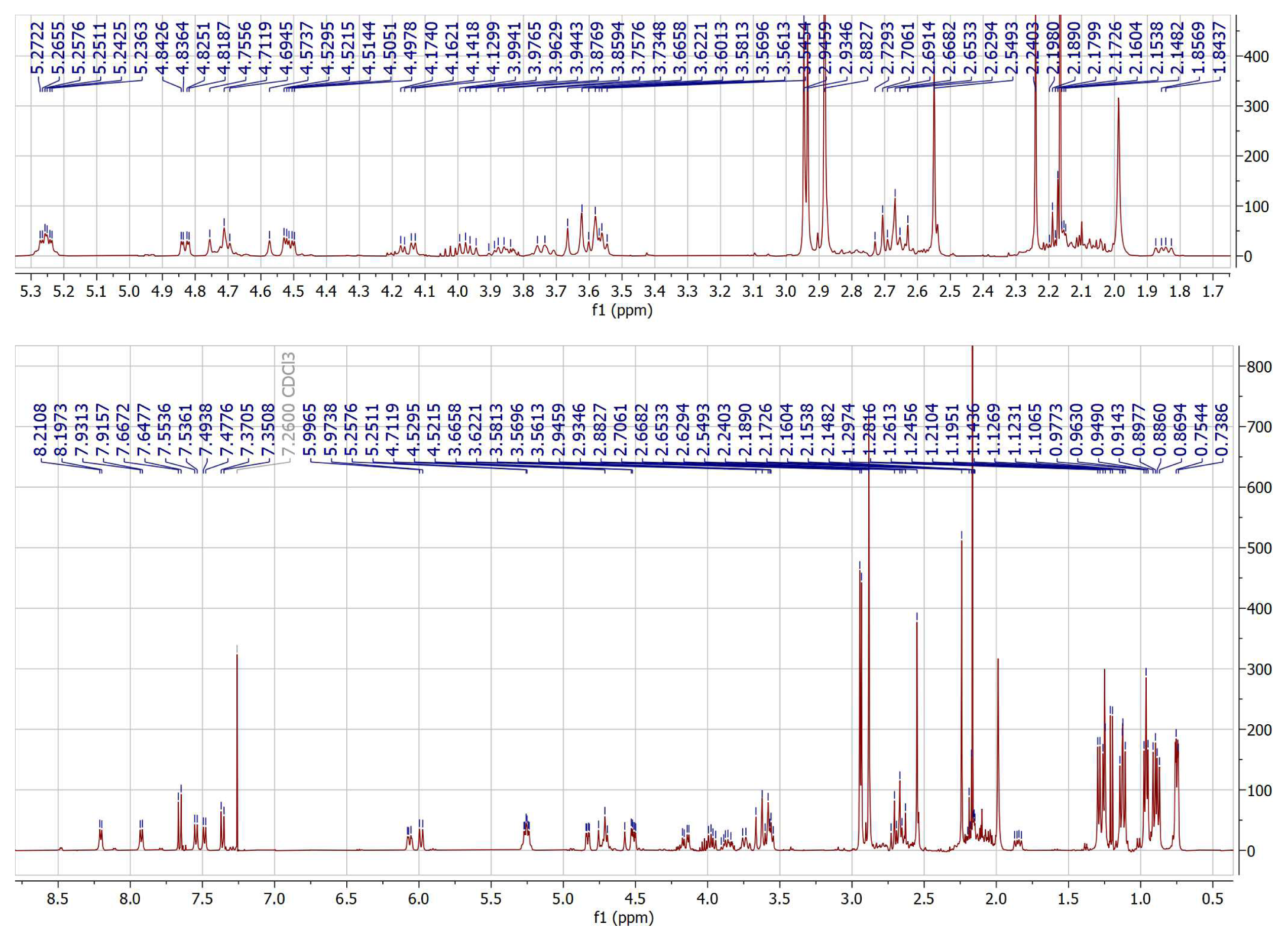

Supplement: Figure S7 — The 1H NMR spectrum of Compound 3 (in CDCl3, 1H: 400 MHz, 13C:100 MHz). [file tjb-50-01-17s7.tif]

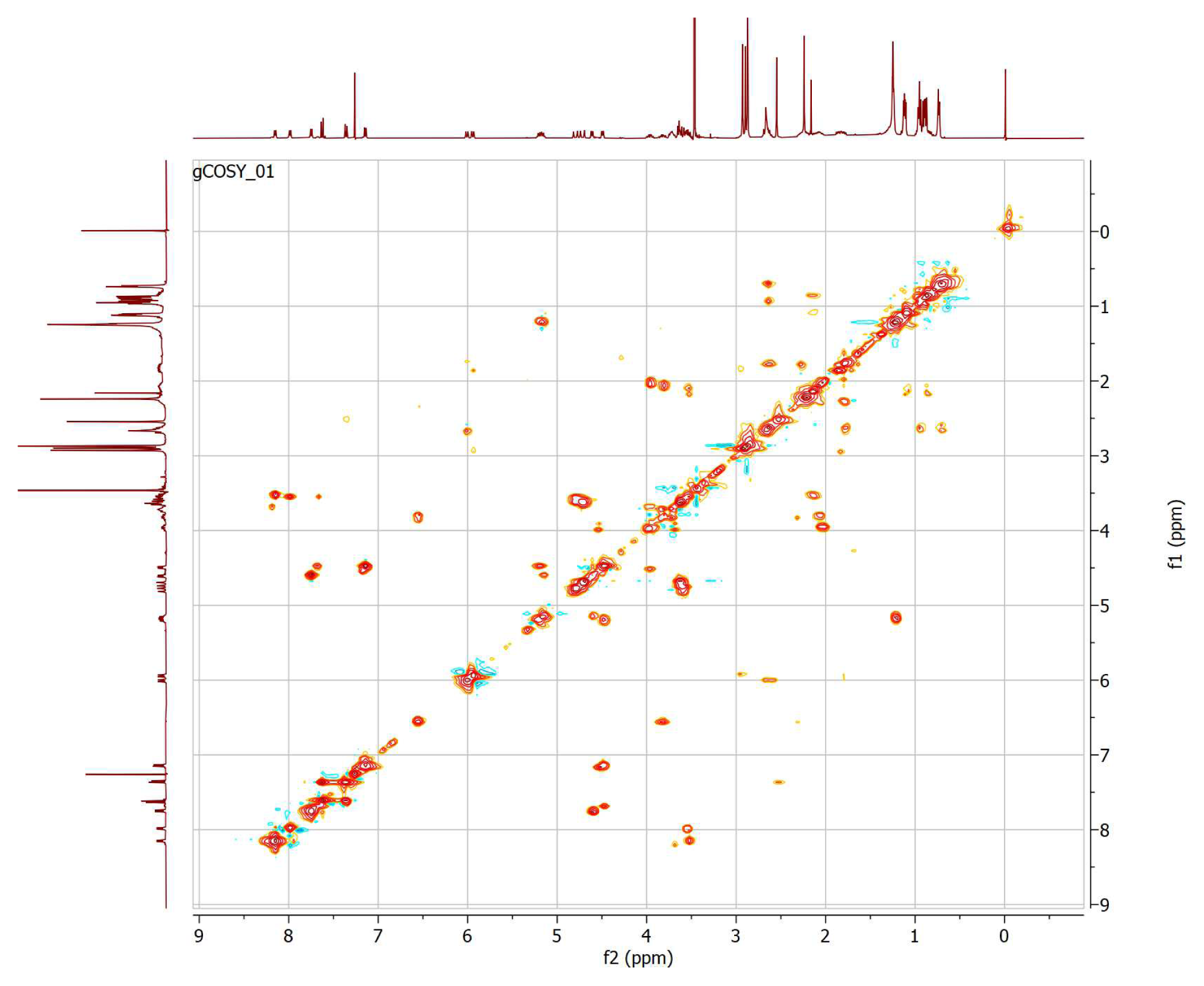

Supplement: Figure S8 — The COSY spectrum of Compound 1 (in CDCl3, 1H: 400 MHz, 13C:100 MHz). [file tjb-50-01-17s8.tif]

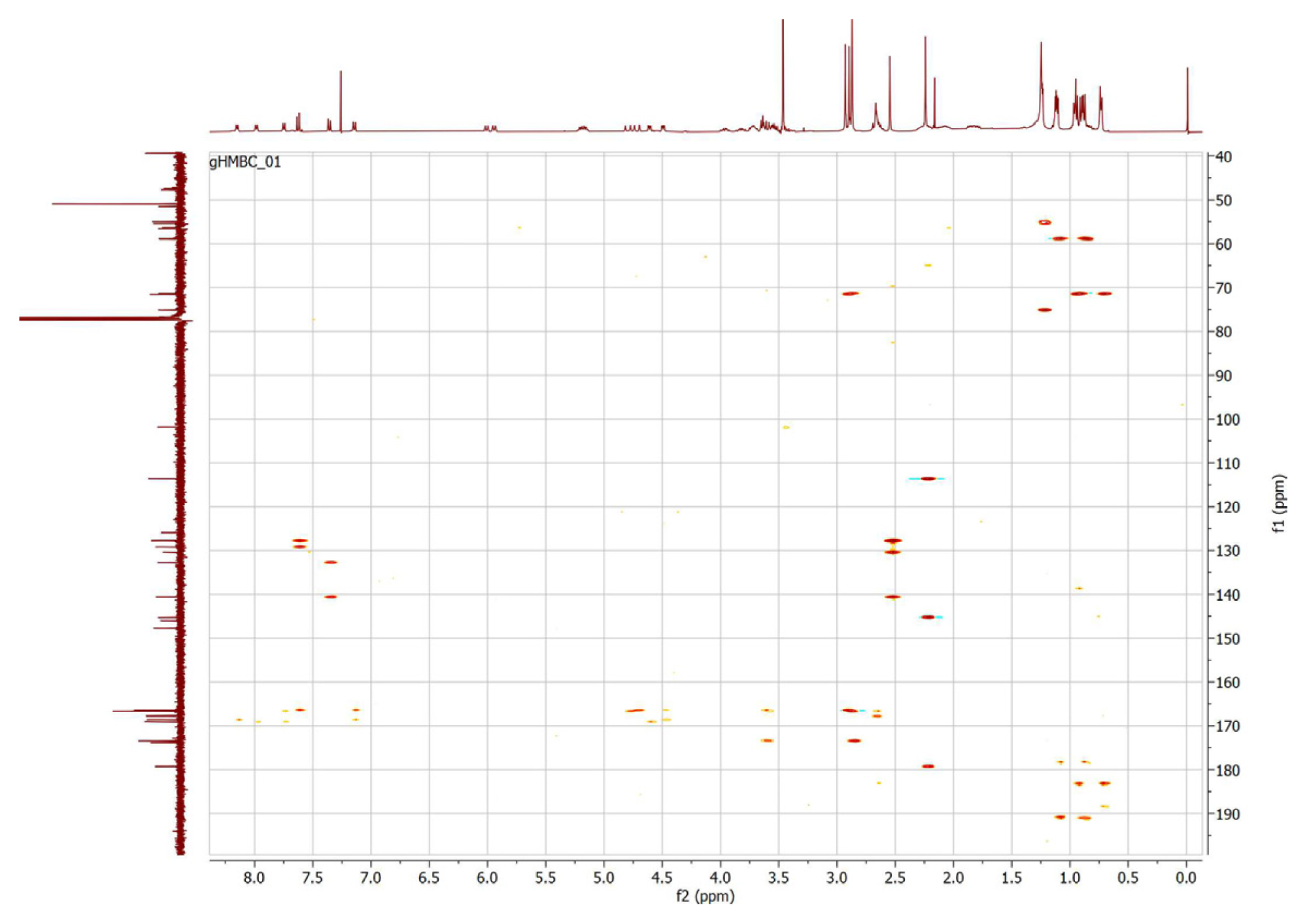

Supplement: Figure S9 — The HMBC spectrum of Compound 1 (in CDCl3, 1H: 400 MHz, 13C:100 MHz). [file tjb-50-01-17s9.tif]

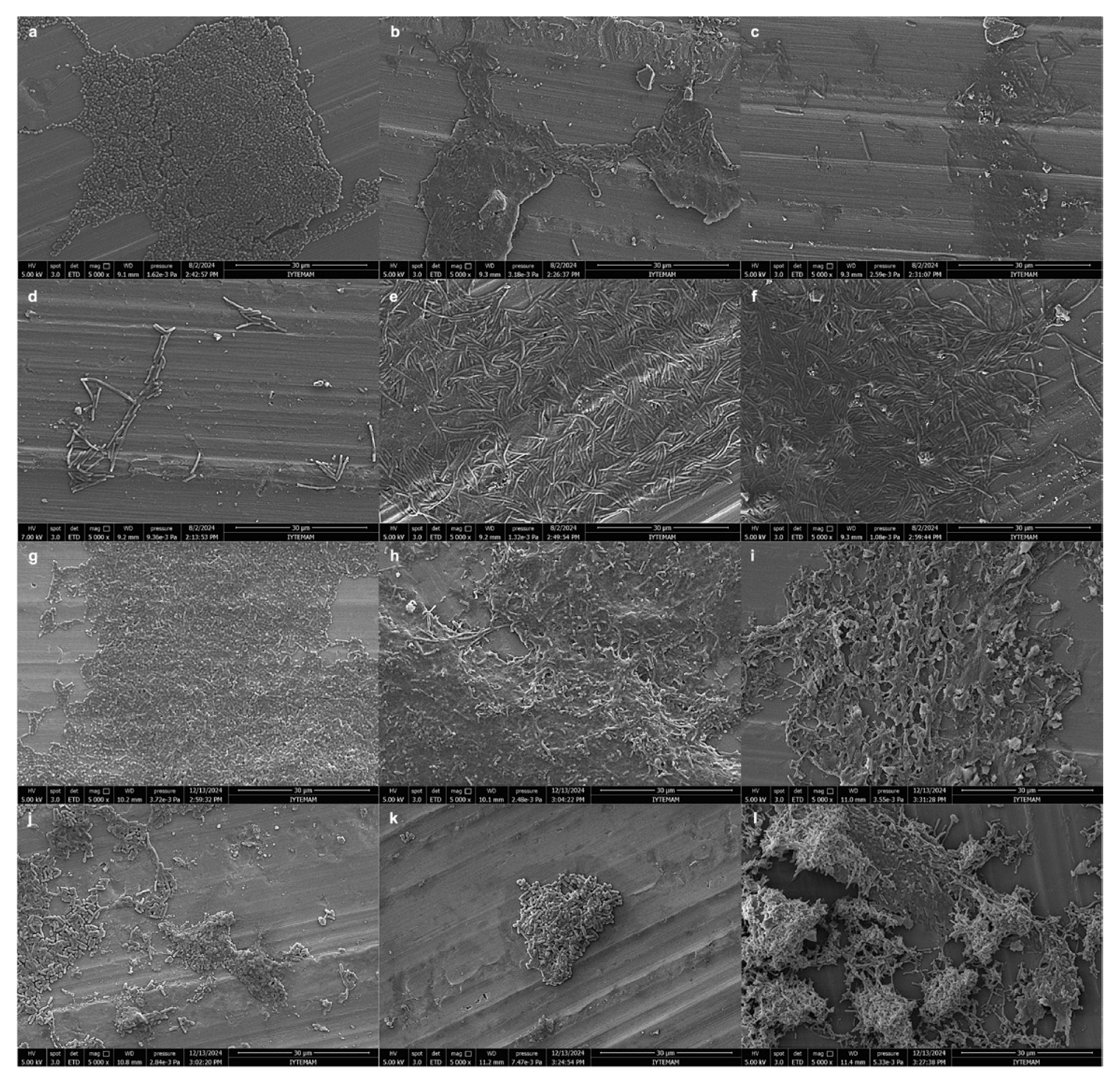

Supplement: Figure S10 — The changes in the morphology of E. coli at 5000 × magnification in SEM upon the individual treatment at MICs and combinations at MSCs; a) vehicle control, b) actinomycin D, c) actinomycin X2, d) nalidixic acid, e) combination of actinomycin D: nalidixic acid, f) combination of actinomycin X2: nalidixic acid, g) polymyxin B, h) combination of actinomycin D: polymyxin B, i) combination of actinomycin X2: polymyxin B, j) kanamycin, k) combination of actinomycin D: kanamycin, l) combination of actinomycin X2: kanamycin. Scale bars (30 μm) are shown at the lower right of each panel. [file tjb-50-01-17s10.tif]
